# Complete atrioventricular dissociation and sinus arrest after pheochromocytoma resection

**DOI:** 10.1002/iju5.12160

**Published:** 2020-04-18

**Authors:** Yuya Yamada, Hiroshi Fujiwara, Haruka Banno, Kensuke Hikami, Masakazu Nakashima, Masahiro Tamaki, Noriyuki Ito

**Affiliations:** ^1^ Urology Department Japanese Red Cross Wakayama Medical Center Wakayama Japan

**Keywords:** bradycardia, complete atrioventricular dissociation, pheochromocytoma, sinus arrest, vagal reflex

## Abstract

**Introduction:**

Although preoperative bradycardia has been reported in several pheochromocytoma cases, postoperative bradycardia has not. This is the first case report of complete atrioventricular dissociation and sinus arrest occurring after pheochromocytoma resection.

**Case presentation:**

A 38‐year‐old woman was referred for a left adrenal incidentaloma. Twenty‐four hour urinary collection showed elevated noradrenaline. Iodine‐123‐meta‐iodobenzylguanidine scintigraphy showed high tracer uptake in the left adrenal region. Open left adrenalectomy was performed, and histopathological examination confirmed the diagnosis of pheochromocytoma. Thirty minutes following surgery, complete atrioventricular dissociation and sinus arrest developed. Vagal reflex attenuation due to decreased noradrenaline after tumor removal and perioperative pain and fear were believed to be the causes. A temporary pacemaker was implanted to prevent sudden death due to vagal overstimulation.

**Conclusion:**

Vagal reflex attenuation after pheochromocytoma resection can result in complete atrioventricular dissociation and sinus arrest. Adequate preoperative preparation and close monitoring during and after surgery are imperative.

**Keynote message**

We report the case of a 38‐year‐old woman with complete atrioventricular dissociation and sinus arrest occurring after pheochromocytoma resection. Vagal reflex attenuation due to decreased noradrenaline after tumor removal and perioperative pain and fear were believed to be the causes. Adequate preoperative preparation and close monitoring during and after surgery are imperative.

## Introduction

Pheochromocytomas are catecholamine‐producing neuroendocrine tumors arising from chromaffin cells of the adrenal medulla or extra‐adrenal paraganglia.^1^ Secreted catecholamines can potentially cause lethal cardiovascular complications and arrhythmias. While patients with catecholamine‐secreting tumors usually exhibit tachycardia during the florid phase of their illness, reports have shown that 10% of the patients develop bradycardia.^2^ Although a number of studies have shown that some patients with pheochromocytoma present with bradycardia during the preoperative period,^3^, ^4^, ^5^, ^6^, ^7^, ^8^ postoperative bradycardia has remained largely unknown. We present a patient who developed complete atrioventricular dissociation and sinus arrest in the postoperative period after pheochromocytoma resection, which prompted temporary pacemaker implantation.

## Case presentation

A 38‐year‐old woman was referred to our department for a large left adrenal incidentaloma. She had a history of intractable headache and recent left abdominal pain and swelling. The family history revealed that her aunt was diagnosed with gastric cancer. Physical examination showed blood pressure 147/98 mmHg, height 166 cm, weight 57 kg, and body mass index 20.6. Electrocardiography and chest X‐ray findings were normal. Routine laboratory findings were within the physiologic range.

**Fig. 1:**
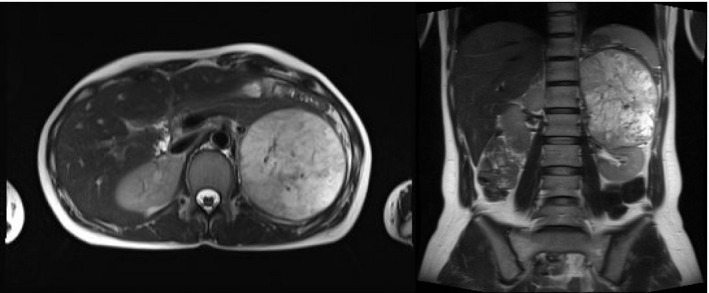
Abdominal magnetic resonance imaging revealing a large 11 × 8.5 cm left adrenal tumor.

**Fig. 2:**
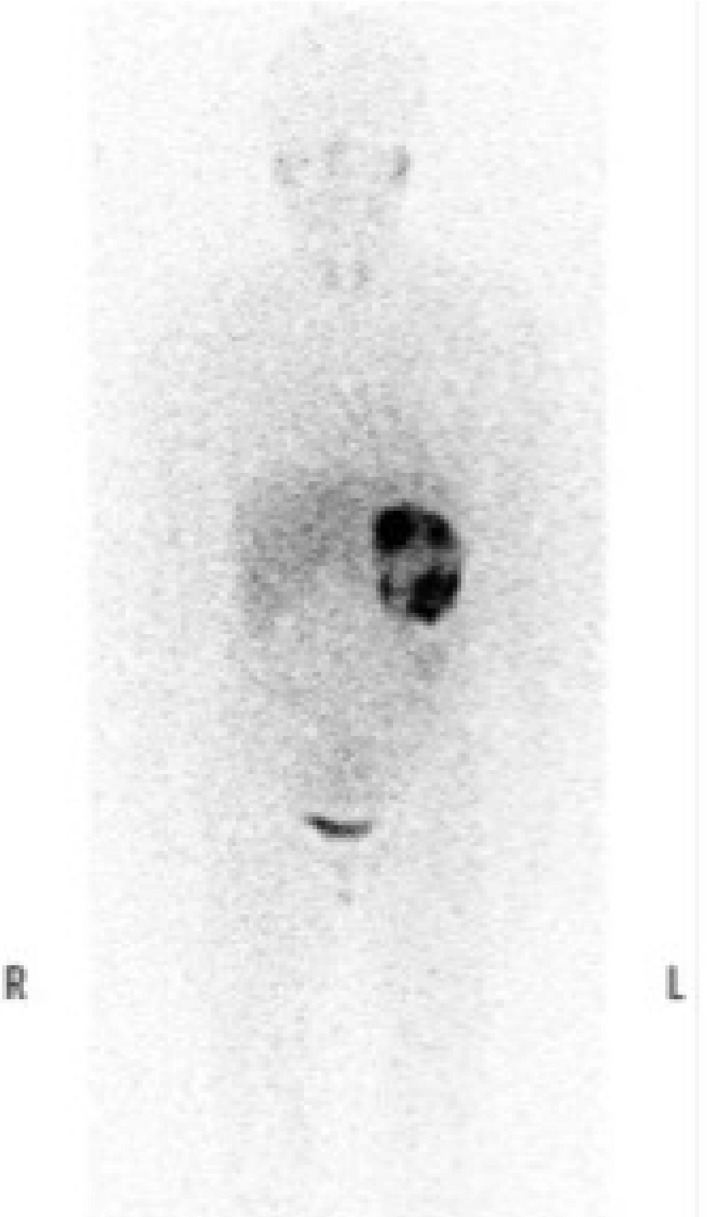
Iodine‐123‐meta‐iodobenzylguanidine scintigraphy showing high tracer uptake in the left adrenal region.

High‐performance liquid chromatography on 24‐h urinary collections showed elevated noradrenaline (863.7 μg/day, normal range 48–168 μg/day), normetanephrine (19.8 mg/day, normal range 0.09–0.33 mg/day), and vanillylmandelic acid (43.1 mg/day, normal range 1.5–4.3 mg/day). An abdominal magnetic resonance imaging revealed a large 11 × 8.5‐cm left adrenal tumor (Fig. 1), while iodine‐123‐meta‐iodobenzylguanidine scintigraphy showed high tracer uptake in the left adrenal region (Fig. 2).

**Fig. 3:**
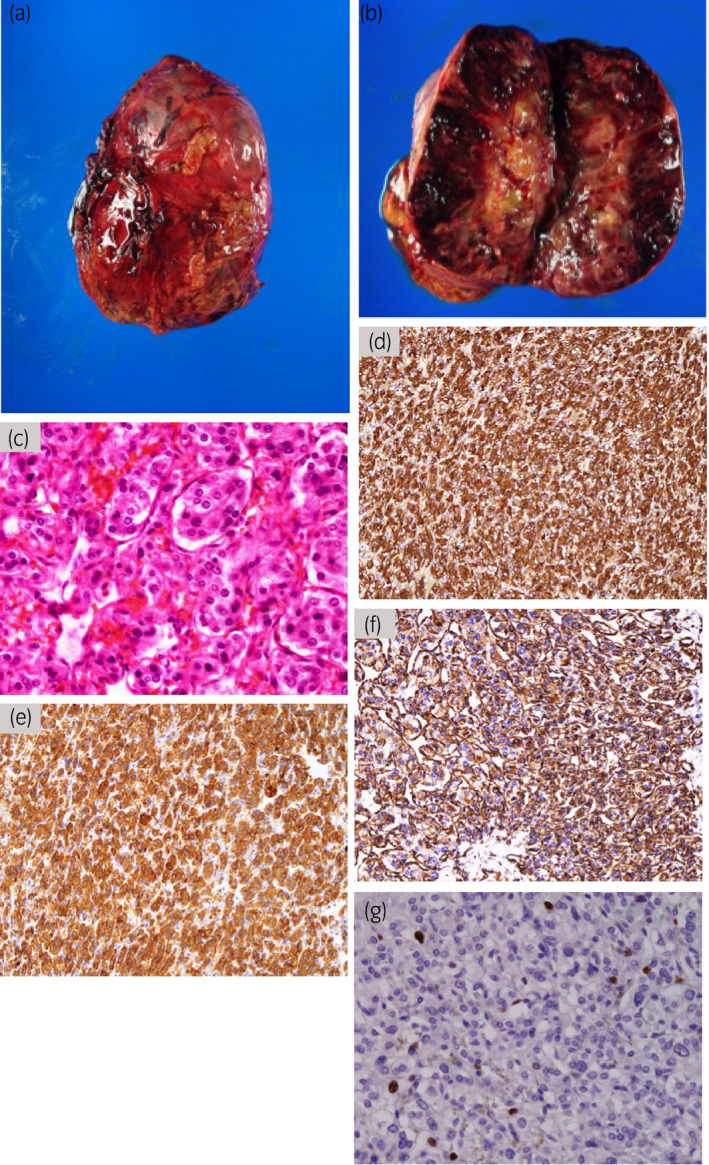
The tumor was an ovoid, tan‐red, soft tissue measuring 11 × 8.5 cm (a). Sectioning of the specimen showed a yellowish‐white, firm, well‐circumscribed mass with red‐brown hemorrhage (b). Hematoxylin and eosin stain showed polygonal cells arranged in large nests surrounded by a sustentacular framework with central tumor necrosis (c). Immunohistochemically, the tumor was positive for chromogranin A (d), synaptophysin (e), and vimentin (f). The Ki67 index was 3.7% (g). The Grading System for Adrenal Pheochromocytoma and Paraganglioma score was 7 based on large nests, high cellularity, necrosis, and Ki67 index >3%.

Pretreatment with the alpha‐adrenergic blocker doxazosin once a day at a dose increasing from 1 to 8 mg for 3 weeks and saline infusion of 1000 mL/day for 2 days had been performed before surgery. Open left adrenalectomy was performed 8 weeks after initial consultation through a modified Makuuchi incision. The surgical time was 360 min, and the blood loss was 673 mL. Histopathological examination confirmed the diagnosis of pheochromocytoma (Fig. 3).

**Fig. 4:**
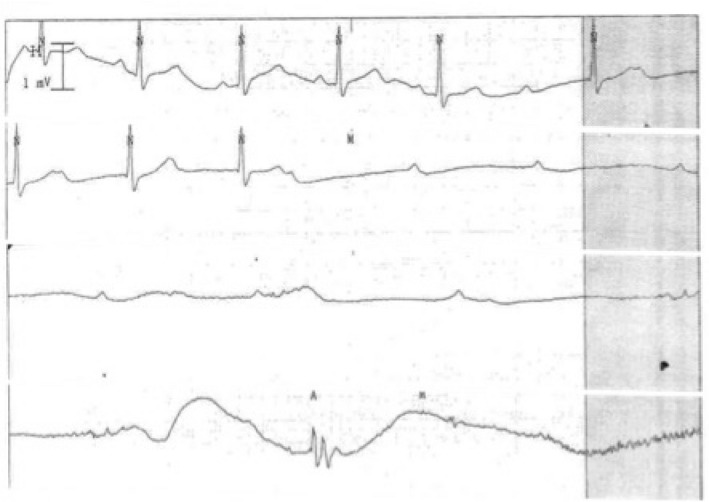
Electrocardiography showed a gradual decrease in the sinus rate (prolonged P–P interval) and atrioventricular conduction (prolonged P–R interval), followed by complete atrioventricular dissociation and sinus arrest.

The postoperative condition of the patient was monitored in the intensive care unit. In the initial evaluation of her postoperative condition, sustained hypotension after tumor removal despite intravenous infusion of noradrenaline was found, while cardiosonography showed no wall motion abnormalities. Thirty minutes after surgery, the patient experienced nausea and vomiting, and subsequently complete atrioventricular dissociation and sinus arrest developed (Fig. 4). After cardiopulmonary resuscitation was initiated, sinus rhythm was immediately observed. However, complete atrioventricular dissociation and sinus arrest secondary to nausea recurred repeatedly at intervals of several minutes, and therefore a temporary pacemaker was implanted. Although the temporary pacemaker functioned twice within 24 h after implantation, bradycardia was not observed in the 24 h that followed. The temporary pacemaker was therefore removed on postoperative day 2, and the patient was discharged on postoperative day 15 without any sequelae. Follow‐up examination showed no evidence of tumor recurrence. Since discharge, no episodes of symptomatic bradycardia or syncope have been observed.

## Discussion

A number of studies have reported that patients with pheochromocytoma developed bradycardia, including sinus arrest or atrioventricular dissociation, during the preoperative period,^3^, ^4^, ^5^, ^6^, ^7^, ^8^ among whom tumor removal preceded arrhythmia resolution. Excess catecholamine secretion increased vagal discharge through the baroreceptor reflex, and adrenergic receptor desensitization have been considered causes of bradycardia.

In the present case, although no electrocardiographic changes were observed during preoperative examination, complete atrioventricular dissociation and sinus arrest developed after tumor resection. Bradycardia and conduction disorder are often due to cardiomyopathy, myocardial inflammation or infarction, and metabolic disorder (hyper‐ or hypokalemia, hypothermia, hypothyroidism, and hypoxia);^9^ however, none of these mechanisms were observed in the present case. The coexistence of complete atrioventricular dissociation and sinus arrest indicated simultaneous sinus node depression and atrioventricular nodal conduction, suggesting that the parasympathetic system (vagus nerve) above the two nodes might be involved.^10^ Vagal activation is more common in young females^11^ and is triggered by reduced cardiac venous return and by affective mechanisms, such as pain and fear.^12^ In the present case, the patient may have been prone to vasovagal activation related to her age and gender. The noradrenaline‐induced depletion of intravascular volume and also the peripheral vasodilation and subsequent reduction in cardiac venous return caused by the sudden decrease in noradrenaline concentration after tumor removal led to vagal activation. Intraoperative hemorrhage, as well as postoperative pain and fear, can also be considered contributing factors. Finally, these factors caused vagal overstimulation leading to recurrent sinus arrest. Nausea preceding bradycardia was considered to be prodromic.

Although vagally mediated death is rare, some deaths can be attributed to vagal overstimulation.^10^, ^13^ To prevent sudden death from vagal overstimulation, the patient underwent temporary pacemaker implantation, considering that permanent pacemakers have not been recommended for vagally mediated bradycardia, especially in young patients.^10^, ^14^ After the patient had recovered from hypovolemia and episodes of bradycardia, the temporary pacemaker was removed.

Some limitations of this case report are worth noting. First, we were unable to validate the effectiveness of atropine and ephedrine, which have been reported to be effective for vagally mediated bradycardia and sinus arrest.^11^ Second, considering that vagal reflex generally recovers spontaneously, complete atrioventricular dissociation and sinus arrest might have resolved without resuscitation. However, this could not be confirmed.

Nevertheless, adequate preoperative preparation with alpha‐adrenergic blockers and increased salt and fluid intake to prevent postoperative reduction in cardiac venous return and consequently vagal activation, and close monitoring during and after surgery, are imperative.

## Conclusion

To the best of our knowledge, this is the first case report of complete atrioventricular dissociation and sinus arrest occurring after pheochromocytoma resection. Vagal reflex attenuation due to decreased noradrenaline after tumor removal and perioperative pain and fear were believed to be the causes. Adequate preoperative preparation to prevent postoperative reduction in cardiac venous return and close monitoring during and after surgery are imperative.

## Conflict of interest

The authors declare no conflict of interest.
